# Surgical Management of Corneal Damage in Patients with Primary and Post-Surgical Eyelid Anomalies

**DOI:** 10.3390/jcm15093331

**Published:** 2026-04-27

**Authors:** Francesco Aiello, Flavia Quaranta Leoni, Luigi Mosca, Rossella Anna Maria Colabelli Gisoldi, Vincenzo Maurino, Carlo Nucci

**Affiliations:** 1 Ophthalmology Unit, Department of Experimental Medicine, University of Rome “Tor Vergata”, 00133 Rome, Italy; francesco.aiello@ptvonline.it (F.A.); carlo.nucci@ptvonline.it (C.N.); 2 Ophthalmology Department, Catholic University “Sacro Cuore”, 00168 Rome, Italy; luigi.mosca@policlinicogemelli.it; 3 Ophthalmology Department, Eye Bank, San Giovanni Addolorata Hospital, 00184 Rome, Italy; ramcolabelligisoldi@hsangiovanni.roma.it; 4 Moorfields Eye Hospital NHS Foundation Trust, London EC1V 2PD, UK; moorfields.vmaurino@nhs.net

**Keywords:** eyelid anomalies, corneal damage, stromal thinning, corneal melt, perforation, tectonic keratoplasty, ocular surface reconstruction

## Abstract

Eyelid anomalies represent a relevant cause of corneal injury, including epithelial instability and recurrent erosions up to progressive stromal thinning, corneal melt, and, in severe cases, perforation leading to permanent visual impairment. Correction of eyelid dysfunction is the first step in managing these lesions. However, corneal damage may persist or progress despite adequate eyelid treatment. Therefore, a corneal surgical approach is necessary to preserve ocular surface integrity and visual function. This review synthesizes literature published between 2008 and 2025 on corneal complications secondary to eyelid anomalies and postoperative eyelid procedures. We analyzed the mechanisms of eyelid-induced corneal injury, indications for surgical treatment, and corneal surgical strategies, from surface-stabilizing techniques to tectonic interventions. Entropion and ectropion are the most common eyelid abnormalities associated with mechanical trauma and exposure-related corneal disease. Although definitive eyelid correction is necessary for corneal recovery, persistent epithelial defects, stromal thinning, corneal melt, and perforation frequently require corneal surgical management. Surface-stabilizing procedures, such as amniotic membrane transplantation, are effective in early disease stages, whereas progressive stromal defects necessitate tectonic approaches such as lamellar patch grafting or therapeutic keratoplasty. Interventions aimed at visual rehabilitation should be postponed until sustained ocular surface stability has been achieved. Effective management of eyelid-related corneal damage requires both eyelid surgical correction and corneal management. Close collaboration between corneal and oculoplastic surgeons helps achieving good anatomical outcomes and long-term ocular surface stability.

## 1. Introduction

The eyelids have a crucial role in preserving corneal integrity and visual function. Proper lid margin apposition, appropriate lash orientation, and adequate blink frequency are key determinants of ocular surface homeostasis [1,2]. Any disturbance in these factors—whether related to age, congenital anomalies, trauma, or iatrogenic causes—can lead to corneal injury [2,3]. The tear film itself is a complex, multilayered structure consisting of lipid, aqueous, and mucin components [1]. Eyelid malposition that results in incomplete closure, lagophthalmos, or misdirected lashes can destabilize the tear film, leading to desiccation, punctate epithelial erosions, and increased susceptibility to infection [1,2]. If untreated, exposure keratopathy may progress rapidly, particularly in older adults or in those with pre-existing ocular surface disease [2,4,5,6]. Corneal injury secondary to eyelid anomalies presents a broad spectrum of clinical manifestations. Mild epithelial disruptions may be asymptomatic or cause irritation, tearing, and foreign body sensation. More pronounced injuries can lead to stromal thinning, recurrent erosions, or superficial ulceration, while severe trauma may progress to stromal necrosis, infection, or perforation [2,7,8]. Visual prognosis depends on the location, depth, and chronicity of the lesion, with central corneal involvement posing the highest risk for permanent visual impairment [2,9]. This underlines the necessity of early diagnosis and intervention [7,9]. Beyond the immediate threat to vision, corneal damage resulting from eyelid pathology carries functional and psychological consequences, frequently associated with discomfort, photophobia, and reduced quality of life [9,10]. Although conservative therapies—such as lubricating drops, ointments, or temporary tarsorrhaphy—can provide temporary relief, definitive management of eyelid-related corneal damage requires surgical correction [7,9]. The choice of technique depends on the underlying pathophysiology, the severity of corneal compromise, and patient factors [2]. Surgical goals include restoring eyelid position, reestablishing proper lid margin apposition and lash orientation, stabilizing the ocular surface to facilitate epithelial healing, and employing ocular surface reconstruction when necessary [2,7,11,12,13,14,15]. Advances in oculoplastic surgery and ocular surface reconstruction have significantly improved functional and cosmetic outcomes while minimizing complication rates [4,5,11,12]. Incorporating evidence from recent literature allows surgeons to tailor interventions to each patient’s anatomical and functional needs [9]. This narrative review gives an overview of the surgical management of corneal damage associated with eyelid anomalies, synthesizing clinical studies, case series, and reviews published between 2008 and 2025. This narrative review examines the role of eyelid-related factors in corneal damage, highlighting both primary etiologies and those arising secondary to eyelid surgery, and provides a comprehensive overview of current ocular surface reconstruction techniques [3,7,8,16,17,18,19]. By consolidating evidence on surgical methods, outcomes, and complications, this review aims to guide clinicians in optimizing corneal protection and overall patient care [2,9].

## 2. Materials and Methods

This study was designed as a narrative review with a structured literature search aimed at synthesizing current evidence on corneal damage secondary to eyelid anomalies and related surgical management strategies.

A comprehensive literature search was conducted using PubMed/MEDLINE, Scopus, and Web of Science databases, covering publications from January 2008 to March 2025. Seminal articles published before 2008 were also included when considered essential for historical context and understanding of current clinical practice. The search strategy combined keywords and MeSH terms related to both eyelid pathology and corneal complications, including: “eyelid malposition,” “entropion,” “ectropion,” “blepharoplasty complications,” “ptosis surgery,” “ocular surface disease,” “corneal melt,” “stromal thinning,” “corneal perforation,” “amniotic membrane transplantation,” and “tectonic keratoplasty.” Boolean operators (AND/OR) were used to refine the search.

The study selection process was performed by screening titles and abstracts for relevance, followed by full-text evaluation of potentially eligible articles. In addition, the reference lists of selected studies were manually reviewed to identify further relevant publications.

Inclusion criteria comprised clinical studies, case series, case reports, and both narrative and systematic reviews reporting corneal complications associated with eyelid anomalies or eyelid surgical procedures. Exclusion criteria included studies focusing on infectious keratitis, autoimmune-related corneal melting, or ocular surface diseases not related to eyelid pathology. Due to the heterogeneity of study designs, patient populations, and reported outcomes, a quantitative synthesis was not feasible. Therefore, data were analyzed and synthesized qualitatively. A total of 53 articles were included in the final analysis, selected based on their relevance to key aspects of the topic, including: pathophysiological mechanisms of eyelid-related corneal damage; clinical stratification of stromal thinning, corneal melt, and perforation; indications and timing for stepwise surgical escalation; and corneal surgical strategies, ranging from conservative and tissue-preserving approaches (e.g., amniotic membrane transplantation, tissue adhesives) to tectonic and reconstructive procedures (e.g., lamellar and penetrating keratoplasty), with particular emphasis on clinical decision-making and multidisciplinary management.

## 3. Etiology of Corneal Damage

**Figure 1:**
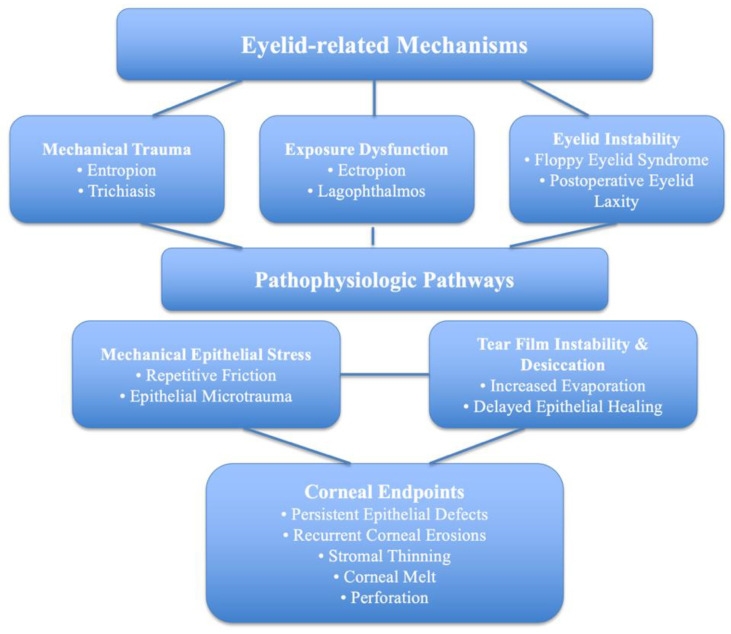
Pathophysiologic mechanisms of corneal damage secondary to eyelid anomalies.

Mechanical trauma, exposure-induced desiccation, or a combination of both are the main mechanisms of eyelid-induced corneal damages [1,2,3,8,9]. The principal pathogenic of these mechanisms are illustrated in Figure 1.

### 3.1. Congenital Eyelid Anomalies

Congenital eyelid abnormalities may predispose patients to early-onset corneal injury. Congenital entropion, distichiasis, and epiblepharon can lead to chronic epithelial irritation through direct lashes contact on ocular surface [20,21,22,23]. Continuous trauma leads to superficial keratopathy, ulceration, or scarring, potentially threatening normal visual development if left untreated and therefore underlining the necessity of early surgical management [21,22].

### 3.2. Eyelid Malposition

In adults, eyelid malposition represents the most common cause of eyelid-related corneal injury [3,20]. Entropion is characterized by inward rotation of the eyelid margin, most commonly of the lower lid. Causes of entropion involves a combination of horizontal lid laxity, attenuation or disinsertion of the lower lid retractors, and overriding of the preseptal orbicularis over the pretarsal portion [20,21,22]. These changes result in misdirected lashes which damage the corneal epithelium, leading to punctate erosions, recurrent ulceration, and, in severe cases, stromal thinning [3]. In literature there is a correlation between disease duration and severity of corneal damage, underlining the importance of early surgical correction [3]. Ectropion is characterized by outward displacement of the eyelid margin, typically due to horizontal laxity, canthal tendon attenuation, or vertical lid shortening [20]. This results in chronic exposure, tear film instability, and punctate epithelial erosions [2,20]. Prolonged exposure causes epithelial lesions, stromal thinning, and ulceration, particularly in older patients or those with ocular surface disease [2,4].

### 3.3. Mechanical Eyelid Disorders

#### 3.3.1. Trichiasis

Trichiasis is defined as misdirection of eyelashes toward the ocular surface with normally positioned eyelid margin. It is classified as a primary condition or secondary to chronic inflammatory eyelid disease, cicatricial disorders, or postsurgical scarring. Continuous lash–cornea contact induces focal epithelial trauma, which causes punctate keratopathy, recurrent erosions, or visually significant corneal scarring in advanced cases [23,24,25].

#### 3.3.2. Floppy Eyelid Syndrome

Floppy eyelid syndrome (FES) is characterized by eyelid laxity, typically involving the upper eyelid, with likelihood of spontaneous eversion during sleep or minimal manipulation. This mechanical instability disrupts blink dynamics and tear film distribution, predisposing the cornea to chronic exposure and repetitive microtrauma [23,24,25]. Clinically, patients may present with punctate epithelial erosions, recurrent epithelial defects, or sterile corneal infiltrates, often with asymmetric involvement related to habitual sleeping position [23]. The association with obstructive sleep apnea further contributes to ocular surface inflammation and delayed epithelial healing [23,24,25].

### 3.4. Iatrogenic Eyelid Pathologies

With the current increase in cosmetic and functional eyelid procedures, iatrogenic causes of corneal injury are being recognized with greater frequency [16,17,18,19]. Oculoplastic interventions may significantly influence ocular surface homeostasis and, in some cases, result in severe corneal complications if postoperative eyelid dynamics are altered or ocular surface protection is insufficient [26,27]. Postoperative mechanisms of corneal damage include lagophthalmos, altered blink dynamics, suture-related trauma, and thermal injury from surgical instrumentation. These factors may compromise tear film stability, promote epithelial disfunction, and predispose the cornea to progressive stromal damage if not promptly recognized and managed [16,17,18,19,28,29,30,31,32]. This is especially critical in corrective eyelid surgeries for pre-existing eyelid anomalies that have already led to corneal damage, as inadequate management may exacerbate an already compromised ocular surface. Common postoperative eyelid surgery-related mechanisms leading to corneal damage are summarized in Table 1.

Upper eyelid procedures may inadvertently disrupt eyelid mechanics, alter blink dynamics, and destabilize the tear film. Documented complications include lagophthalmos, exposure keratopathy, suture-related trauma, and induced corneal astigmatism [4,5,6,7,16,17,18,19]. Suture-related corneal injury following buried-suture double-eyelid blepharoplasty has been increasingly reported and may lead to chronic epithelial defects, stromal inflammation, and delayed diagnosis if not promptly recognized [28,29,30,31]. Although rare, diathermy-related corneal burns may occur during upper eyelid surgery and can rapidly progress to stromal melt if not promptly recognized and managed [32].

### 3.5. Multifactorial and Combined Mechanisms

In clinical practice, corneal damage frequently arises from the interaction of multiple pathogenic mechanisms. For example, entropion combined with postoperative lagophthalmos may simultaneously induce mechanical abrasion and exposure-related desiccation [2,3,21]. Recognition of these overlapping processes is essential for surgical planning and for anticipating the need for corneal surgical intervention beyond eyelid correction alone [13,21].

## 4. Corneal Surgical Management

**Figure 2:**
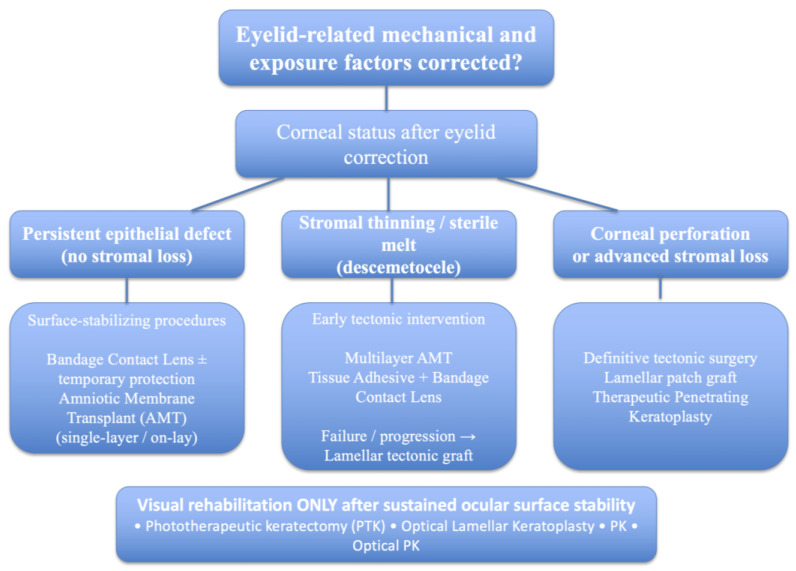
Stepwise Corneal Surgical Management of Eyelid-Related Stromal Thinning, Melt, and Perforation.

The aim of surgical intervention in patients with corneal damage secondary to eyelid anomalies is the preservation of ocular surface integrity and visual function through elimination of pathogenic mechanical and exposure-related factors. The first step in the management of these patients is correcting the underlying cause, hence addressing the eyelid problem. Although conservative measures—including intensive lubrication, therapeutic bandage contact lenses, or temporary tarsorrhaphy—may provide short-term stabilization, they rarely offer definitive resolution when eyelid dysfunction persists [13,21]. Although restoration of normal eyelid anatomy and function is fundamental to remove the primary source of corneal injury, clinical experience indicates that corneal pathology may persist or progress despite adequate eyelid correction [13,21]. Patients presenting with significant epithelial defects, stromal thinning, or recurrent corneal injury should be promptly evaluated in a multidisciplinary setting to coordinate eyelid correction and corneal management [20,21,26,27]. In such cases, corneal reconstructive procedures may be required to restore structural integrity and prevent progression to perforation. These may include lamellar patch grafting, deep anterior lamellar keratoplasty, therapeutic penetrating keratoplasty, or conjunctival flap procedures, depending on the extent of tissue loss and ocular surface stability [33,34,35,36,37]. The primary goals of corneal surgery are restoration of epithelial integrity, provision of tectonic support when stromal compromise is present, and preservation or rehabilitation of visual function [13,20]. Surgical decision-making must account for the depth and extent of corneal involvement, the presence of inflammation or infection, and the likelihood of recurrent surface stress [20,21]. Corneal surgery is indicated in case of persistent epithelial defects refractory to lubrication, bandage contact lenses, or temporary tarsorrhaphy [13,14], progressive stromal thinning or sterile corneal melt [20,21], descemetocele formation or impending perforation [17,18,19,21], frank corneal perforation [20], or visually significant scarring or irregular astigmatism following resolution of acute disease [10]. A persistent epithelial defect (PED) is commonly defined as a failure of corneal epithelial healing lasting ≥14 days despite appropriate conventional medical therapy. Such defects reflect impaired epithelial maintenance and represent a major risk factor for stromal thinning, melt, and secondary perforation if not promptly addressed [13,14]. Early recognition of cases requiring surgical treatment is critical, as delayed intervention may result in irreversible stromal loss or loss of globe integrity [20,21]. A stepwise cornea-centered surgical algorithm guiding this decision-making process is summarized in Table 2 [20,21] and schematically represented in Figure 2.

### 4.1. Biologic and Surface-Stabilizing Surgical Techniques

Amniotic membrane transplantation (AMT) represents a cornerstone of surgical management for epithelial and superficial stromal disease in eyelid-related corneal injury. Its anti-inflammatory, anti-fibrotic, and epithelial-promoting properties make it particularly effective in promoting surface healing once mechanical stress has been reduced [11,12]. AMT may be applied as: single-layer (inlay) grafts for persistent epithelial defects and superficial thinning [13,14,15], on-lay patches to reduce friction and support re-epithelialization [11,12], multilayer constructs for deeper stromal thinning, descemetoceles, or early sterile melt [17,18,19]. In this context, AMT serves both as a biologic scaffold and a temporary tectonic support, often delaying the need for more invasive corneal surgery when combined with adequate eyelid protection [17,18,19,20]. Targeted epithelial debridement of nonadherent edges, removal of necrotic tissue, and optimization of the wound bed may facilitate epithelial closure in selected cases [13,14]. When focal leaks or small perforations (<2 mm) are present, tissue adhesives—most commonly cyanoacrylate—combined with a bandage contact lens can provide immediate sealing and short-term tectonic stability [38,39]. These measures are particularly useful as bridging strategies in eyes requiring staged reconstruction or optimization of eyelid function prior to definitive corneal surgery [20,38]. In eyes with chronic surface inflammation, recurrent epithelial breakdown, or peripheral non–visual-axis involvement, conjunctival advancement flaps or Tenon’s tissue grafts provide durable vascularized coverage [40,41,42]. These techniques suppress ongoing stromal melt and enhance tectonic stability, albeit at the expense of optical clarity. As such, they are reserved for vision-threatening disease where structural preservation takes priority over immediate visual rehabilitation [40,41,42].

### 4.2. Surgical Management of Stromal Thinning, Corneal Melt, and Perforation

Stromal thinning, sterile corneal melt, and perforation represent the most vision-threatening manifestations of eyelid-related corneal injuries [20,21]. These conditions typically arise from persistent mechanical trauma, chronic exposure, or delayed epithelial healing, often compounded by ocular surface inflammation or postoperative factors [2,21]. Surgical management in this setting prioritizes preservation of globe integrity, stabilization of the ocular surface, and prevention of further tissue loss, postponing visual rehabilitation [20]. Once eyelid-related mechanical and exposure factors have been adequately addressed, corneal surgical management should proceed according to the depth, extent, and progression of tissue involvement. Persistent epithelial defects without stromal loss may be managed with surface-stabilizing procedures, whereas progressive stromal thinning or descemetocele formation warrants early tectonic intervention to prevent perforation [13,17,18,19,20,21]. Small focal perforations may be temporarily sealed using tissue adhesives, while larger or unstable defects require definitive lamellar or penetrating keratoplasty to restore globe integrity [20,34,35,43,44,45,46]. Importantly, surgeries for visual rehabilitation should be deferred until sustained ocular surface stability has been achieved [20,47,48]. Corneal melt associated with eyelid anomalies is most commonly sterile in nature and driven by a combination of epithelial breakdown, tear film instability, and enzymatic stromal degradation. This process, also referred to as sterile keratolysis, is characterized by progressive stromal degradation in the absence of active infection and may rapidly progress to descemetocele formation or frank perforation [20,21]. Once stromal thinning progresses beyond the anterior third of corneal thickness, the risk of rapid deterioration and perforation increases substantially [20]. Early surgical intervention is therefore indicated when progressive thinning is documented, even in the absence of frank perforation, particularly in eyes with ongoing exposure or mechanical stress [20,21]. Clinical decision-making must consider not only defect depth and size but also defect location, inflammatory activity, and the feasibility of achieving durable eyelid protection. High-resolution anterior segment optical coherence tomography (AS-OCT) has become a valuable adjunct in this setting, allowing objective quantification of stromal thinning, identification of descemetocele formation, and more accurate assessment of progression, thereby supporting timely surgical decision-making [20,21]. Delayed intervention may convert a potentially manageable thinning process into an emergent full-thickness perforation requiring more invasive surgery [20,34,35]. Multilayer amniotic membrane transplantation (AMT) represents an effective first-line surgical option for moderate-to-severe stromal thinning and descemetocele formation in the absence of active infection [17,18,19]. By providing volumetric support and a biologic scaffold for epithelial migration, multilayer AMT can arrest stromal degradation and promote gradual tissue regeneration [17,18,19]. In eyelid-related damage, multilayer AMT is particularly valuable as it offers immediate tectonic reinforcement [17,18,19], reduces inflammatory mediator activity [15,16], and facilitates epithelial closure once mechanical stress is mitigated [17,18,19]. However, its efficacy is limited in cases of large defects, ongoing exposure, or advanced melt, and careful postoperative monitoring is required to detect early failure [19,20]. For small perforations, less than 2 mm, or impending perforations with focal leakage, tissue adhesives—most commonly cyanoacrylate—combined with a bandage contact lens may provide immediate sealing and short-term tectonic stability [38,39]. These measures are best employed as bridging strategies, allowing time for inflammation control, optimization of eyelid position, or preparation for definitive corneal reconstruction [20,38,39]. Adhesive techniques are generally unsuitable for perforations larger than 2 mm, irregular stromal loss, or areas subjected to persistent mechanical stress, where failure rates are high and progression to larger perforations is common [38,39]. Lamellar tectonic grafting is indicated when stromal loss exceeds the supportive capacity of biologic measures or when multilayer AMT fails to achieve stability [20,43]. Patch grafts may be applied to peripheral or paracentral defects and can be customized in size and thickness to restore corneal contour and strength [43,44]. Advantages of lamellar tectonic approaches include preservation of host endothelium [43,44], a reduced immunologic risk compared with penetrating keratoplasty [43,44,45,46], and improved postoperative surface stability [43,44]. Evidence from large surgical series has demonstrated favorable anatomical and functional outcomes following deep anterior lamellar keratoplasty and related lamellar transplantation techniques [33,45,46]. These techniques are particularly well suited for eyelid-related disease, where surface instability may persist even after eyelid correction and where minimizing graft-related complications is essential [20,43].

### 4.3. Visual Rehabilitation After Surface Stabilization

Once epithelial integrity is restored and eyelid function is stable, secondary procedures aimed at visual rehabilitation may be considered. These include phototherapeutic keratectomy (PTK) for superficial scarring and recurrent erosion syndromes in stable ocular surfaces [47,48], optical lamellar keratoplasty for stromal scarring with preserved endothelial function [33,45,46], optical penetrating keratoplasty for central full-thickness scarring or endothelial compromise [34,35]. Importantly, optical rehabilitation should be deferred until sustained surface stability is achieved, as premature intervention in an unstable ocular surface carries a high risk of recurrence and graft-related complications [20,47,48].

### 4.4. Limbal Stem Cell–Based Approaches

Limbal stem cell deficiency may occur due to persistent mechanical and inflammatory insult. In cases of advanced surface failure due to limbal stem cell deficiency specific approaches may be considered [49,50]. These include autografts, such as conjunctival-limbal autograft (CLAU), simple limbal epithelial transplantation (SLET), autologous cultivated limbal epithelial transplantation (auto-CLET) and allografts, including conjunctival-limbal allograft (CLAL) from living donor, keratolimbal allograft (KLAL) from cadaver and allogenic cultivated limbal epithelial transplantation (allo-CLET) [49,50,51,52]. Such interventions require strict control of eyelid-related stressors and careful patient selection to ensure long-term epithelial maintenance [49,50,51,52].

### 4.5. Postoperative Management and Follow-Up

Effective postoperative management is essential for a durable ocular surface stability after eyelid correction and/or corneal surgery. Inadequate surface protection may result in recurrent epithelial defects, progressive thinning, or failure of corneal reconstruction [13,20,21]. Early management focuses on epithelial protection, lubrication, and rapid detection of complications. Preservative-free tears and ointment should be used frequently, and nighttime lubrication is crucial in patients with residual exposure [1,2,21]. Bandage contact lenses reduce friction and support re-epithelialization, while temporary tarsorrhaphy can be considered in high-risk eyes or after tectonic procedures [13,20]. Close follow-up is recommended to detect epithelial defects, infections, suture-related trauma, or recurrent exposure [16,17,18,19,28,29,30,31,32]. Tear film instability and dry eye are common and may require ongoing lubrication, punctal occlusion, and/or anti-inflammatory therapy [1,2,6,7]. Patients should be educated to report early symptoms (foreign body sensation, photophobia, tearing, or fluctuating vision), before stromal compromise develops [21]. Recurrent malposition may need revision surgery [20,21,22]. Suture-related corneal trauma should be managed with suture removal and surface protection [28,29,30,31]. Persistent exposure may necessitate intensified lubrication, moisture strategies, or temporary tarsorrhaphy, with definitive surgical correction when structural issues persist [13,21]. Refractory epithelial defects or progressive thinning may require repeat amniotic membrane transplantation and, when indicated, escalation to tectonic reconstruction [15,16,17,18,19,20]. Postoperative visual fluctuation may also be influenced by changes in corneal curvature and biomechanical properties following eyelid surgery [53].

## 5. Discussion

Eyelid-induced corneal damage represents a clinically significant cause of ocular surface disease, but is frequently underrecognized [2,21]. The literature reviewed in this article highlights that correction of eyelid anomalies is often insufficient to guarantee corneal recovery if epithelial instability or stromal compromise has already developed [13,20]. Considering etiologies, entropion and ectropion are the most prevalent causes, particularly in elderly patients, while iatrogenic eyelid-related complications are be-coming more frequent with the increasing of cosmetic and functional eyelid surgery [3,4,5,6,7,16,17,18,19,20,21,22,26,27]. The evidence supports the need for a stepwise surgical approach guided by the extension, location, and progression of corneal involvement [13,20]. Early and intermediate management involves surface-stabilizing interventions, particularly amniotic membrane transplantation, which helps epithelial healing, reduces inflammation, and provides temporary tectonic support [15,16,17,18,19]. However, when stromal thinning progresses or fails to respond to biologic measures, lamellar tectonic grafting or therapeutic keratoplasty is required to preserve globe integrity [20,43,44,45,46]. In this context, corneal surgery primarily has a globe-preserving function, with visual rehabilitation postponed until ocular surface stability has been achieved [20,47,48]. The timing of intervention is critical in affecting the outcome [20]. If treatment is delayed, it may lead to corneal melt or perforation, worsening prognosis and increasing surgical complexity [20,26]. On the other hand, optical surgery performed too early with persistent surface instability is associated with high rates of epithelial failure, graft complications, and recurrence [20,47,48]. As highlighted in the literature, successful management depends on close interaction between corneal and oculoplastic surgeons [2,21]. Equally important are postoperative care and close follow-up, which play a decisive role in protecting corneal reconstructions and preventing recurrence [13,20,21]. Future developments will likely improve surgical strategies for eyelid-related disease. Bioengineered alternatives to human amniotic membrane and advances in regenerative and stem cell–based therapies may improve epithelial restoration and tectonic stability in advanced ocular surface failure [16,49,50,51,52]. Advances in anterior segment imaging, including high-resolution optical coherence tomography, already facilitate objective assessment of stromal integrity and may improve timing of intervention [20]. However, current evidence is largely derived from retrospective series and single-center experiences. Further studies are needed to develop accurate algorithms, compare reconstructive strategies, and define long-term outcomes in this complex patient population. Overall, current evidence supports a severity and time-based corneal surgical management integrated with appropriate eyelid correction as the most effective strategy for preventing irreversible corneal damage and preserving visual function in patients with eyelid-related ocular surface disease [20,21].

## 6. Conclusions

Corneal damage secondary to eyelid anomalies progresses from epithelial instability to stromal thinning, corneal melt, and perforation [2,13,20]. Effective management not only requires the correction of eyelid malposition, but also a surgical strategy tailored to the severity and progression of tissue involvement [20,21]. It is essential early recognition of eyelid-related corneal damage, timely intervention on mechanical and exposure-related factors, and appropriate corneal surgical strategies to prevent irreversible damage [13,20]. Surface-stabilizing procedures, tectonic reconstruction, and, if necessary, visual rehabilitation corneal surgery play complementary roles in preserving globe integrity and visual function [15,16,17,18,19,20,43,44,45,46]. Equally important is postoperative care, including close monitoring, management of complications, and patient education, all of which are critical to maintaining long-term ocular surface stability [13,20,21]. Optimal outcomes are achieved through close collaboration between corneal and oculoplastic specialists [2,21]. Continued innovation in corneal reconstruction, regenerative therapies, and decision-support technologies, together with robust multicenter clinical evidence, will further refine surgical strategies and improve outcomes for patients affected by this complex and vision-threatening condition [16,20,49,50,51,52].

## Figures and Tables

**Table 1:** Postoperative complications following eyelid surgery that may result in corneal damage, with underlying mechanisms, corneal manifestations, and clinical considerations.

Type of Eyelid Surgery	Postoperative Complication	Underlying Mechanism	Corneal Manifestations	Clinical Considerations
Upper eyelid blepharoplasty	Lagophthalmos	Overcorrection, excessive skin or muscle resection	Exposure keratopathy, punctate epithelial erosions, persistent epithelial defects	Often transient but may require lubrication, taping, or temporary tarsorrhaphy
Upper eyelid blepharoplasty	Suture-related trauma	Suture exposure or extrusion	Recurrent epithelial erosions, corneal abrasion, focal ulceration	Requires early identification and prompt suture removal
Upper eyelid blepharoplasty	Thermal injury	Diathermy-induced heat transmission	Epithelial defect, stromal thinning, corneal melt	Early recognition critical; may require multilayer AMT
Ptosis surgery	Incomplete eyelid closure	Altered eyelid height or blink dynamics	Exposure keratopathy, tear film instability	Risk increased in patients with pre-existing dry eye disease
Ptosis surgery	Altered corneal biomechanics	Changes in eyelid pressure and corneal curvature	Induced astigmatism, visual fluctuation	Often transient; persistent cases may require refractive evaluation
Lower eyelid surgery	Postoperative ectropion or retraction	Inadequate horizontal or vertical support	Exposure keratopathy, inferior punctate keratitis	May necessitate revision surgery or spacer grafting
Buried-suture double-eyelid surgery	Suture erosion	Foreign body contact with ocular surface	Chronic irritation, epithelial defects, stromal inflammation	Frequently misdiagnosed; requires early intervention
Any eyelid surgery	Delayed blink recovery	Edema or orbicularis dysfunction	Tear film instability, superficial keratopathy	Usually self-limiting; supportive therapy recommended

**Table 2:** Stepwise Corneal Surgical Management of Eyelid-Related Stromal Thinning, Melt, and Perforation.

Corneal Condition	Clinical Features	Primary Surgical Objective	Preferred Corneal Surgical Approach
Persistent epithelial defect	Non-healing epithelial loss despite lubrication and eyelid correction	Restore epithelial integrity	Single-layer amniotic membrane transplantation (inlay or onlay)
Superficial stromal thinning	Stromal loss without descemetocele; stable ocular surface	Prevent progression and support epithelialization	Multilayer amniotic membrane transplantation
Progressive stromal thinning	Increasing stromal loss despite AMT or conservative measures	Provide durable tectonic support	Lamellar tectonic graft or corneal patch graft
Descemetocele	Near full-thickness stromal loss with intact Descemet membrane	Prevent imminent perforation	Multilayer AMT ± lamellar patch graft
Small perforation (<2 mm)	Focal leak with limited tissue loss	Immediate globe sealing	Tissue adhesive (cyanoacrylate) + bandage contact lens
Moderate-to-large perforation	Full-thickness defect with significant tissue loss	Restore globe integrity	Therapeutic lamellar or penetrating keratoplasty
Extensive stromal melt	Rapid tissue loss with poor surface stability	Globe preservation	Therapeutic penetrating keratoplasty
Peripheral non–visual axis disease	Chronic melt or thinning outside the optical zone	Long-term tectonic stability	Conjunctival advancement flap or vascularized tissue coverage
Stable corneal scar	Healed surface with visually significant opacity	Visual rehabilitation	PTK, optical lamellar keratoplasty, or optical penetrating keratoplasty
Refractory ocular surface failure	Recurrent epithelial breakdown; features of limbal stem cell dysfunction	Restore epithelial maintenance	Limbal stem cell–based reconstruction (selected cases)

## Data Availability

No new data were created or analyzed in this study. Data sharing is not applicable to this article.

## Abbreviations

The following abbreviations are used in this manuscript:

FES	Floppy Eyelid Syndrome
AMT	Amniotic Membrane Transplantation
AS-OCT	Anterior Segment Optical Coherence Tomography
PTK	Phototherapeutic Keratectomy
CLAU	Conjunctival-limbal Autograft
SLET	Simple Limbal Epithelial Transplantation
Auto-CLET	Autologous Cultivated Limbal Epithelial Transplantation
CLAL	Conjunctival-limbal Allograft
KLAL	Keratolimbal Allograft
Allo-CLET	Allogenic Cultivated Limbal Epithelial Transplantation
